# MR imaging features of pancreatic schwannoma: a Chinese case series and a systematic review of 25 cases

**DOI:** 10.1186/s40644-021-00390-x

**Published:** 2021-02-15

**Authors:** Zhenshan Shi, Dairong Cao, Qian Zhuang, Ruixiong You, Xiumei Li, Zhongmin Li, Yueming Li, Xinming Huang

**Affiliations:** 1 Department of Radiology, The First Affiliated Hospital of Fujian Medical University, 20 Cha-Zhong Road, Fuzhou, 350005 Fujian China; 2 Department of Pharmacy, Fujian Medical University Union Hospital, 29 Xin-Quan Road, Fuzhou, Fujian China; 3 Department of Radiology, Fujian Medical University Union Hospital, 29 Xin-Quan Road, Fuzhou, Fujian China

**Keywords:** Pancreatic neoplasm, Pancreas, schwannoma, Pancreas, MRI

## Abstract

**Background:**

There is a paucity of existing literature centering on the magnetic resonance (MR) imaging features of pancreatic schwannomas, due to the neoplasm’s nonspecific presentation and its rarity. We aimed to identify the characteristic imaging features of pancreatic schwannoma.

**Methods:**

This retrospective search was conducted for histologically confirmed pancreatic schwannoma in multi-institutional database of pathology. Abdominal magnetic resonance imaging (MRI) was performed before histologic examination and their MR imaging studies were independently reviewed. The search yielded six adults (mean age, 46 years) with a definitive histologic postoperative diagnosis of single pancreatic schwannoma each. Additionally, a comprehensive English and Chinese literature review for pancreatic schwannoma and reported MR-imaging findings since 1961 was also conducted. MR imaging features of those cases in the literature were analyzed, summarized and compared with our case series.

**Results:**

This rare entity appeared to be a well-circumscribed, exophytic, oval or round pancreatic mass with a mean greatest diameter of 3.7 cm. Five schwannomas were located in the pancreatic head-neck and one in the pancreatic tail. On MRI, all cases appeared hypointense on T1-weighted images, inhomogeneous hyperintense on T2-weighted images, and hyperintense on diffusion-weighted images. The mean apparent diffusion coefficient (ADC) values of pancreatic schwannoma were 1.11 ± 0.29 × 10^− 3^ mm^2^/s and significantly lower than the surrounding pancreas. The lesion-to-pancreas signal intensity ratio (SIR) at unenhanced T1-weighted images was 0.53 ± 0.07. On dynamic contrast-enhanced MRI, most of the lesions (67%, 4/6) showed homogeneously iso- or hypointense on arterial and portal venous phases, and hyperenhancement on delayed phase compared with the surrounding pancreas. In our analysis of the time intensity curves, all cases exhibited a gradual enhancement pattern.

**Conclusions:**

A well-circumscribed mass displaying inhomogeneous hyperintensity on T2, marked hypointensity on T1, hyperintensity on DWI, and with early slight enhancement at arterial phase and progressive enhancement at portal venous and delayed phase, may suggest the diagnosis of pancreatic schwannoma.

## Introduction

Pancreatic schwannoma is an extremely rare neoplasm among pancreatic lesions [1, 2]. Pancreatic schwannomas are really significant because they may be easily misdiagnosed as malignant tumors or as other benign neoplasms based on their histologic appearance and imaging features, despite the use of multiple noninvasive imaging modalities [3]. Furthermore, correct diagnosis of a pancreatic schwannoma can lead to an optimal treatment, be helpful in avoiding an unnecessary radical resection, endoscopic ultrasonography or percutaneous biopsies [4–8].

Pancreatic schwannoma is typically a slow-growing, well-encapsulated, benign neoplasm that originated from the epineurium of either the autonomic sympathetic or parasympathetic nerve fibers or the branches of the vagus nerve that travels the pancreas [9]. On microscopic examination, pancreatic schwannomas classically present two distinct patterns of growth: a hypercellular component (Antoni A) and a hypocellular component (Antoni B) [7, 9]. Immunohistochemical labeling is usually needed to make the accurate diagnosis. Strong positive staining for S-100 on immunohistochemical examination is a major marker to make the final diagnosis of pancreatic schwannoma [3, 10–12]. Benign types accounts for 65% of all neurogenic neoplasms, but, up to 15% are malignant transformation. Most pancreatic schwannomas with von Recklinghausen disease have a high risk for the development of a malignancy [11, 13].

There is a paucity of existing literature centering on the imaging features of pancreatic schwannomas, due to the neoplasm’s nonspecific presentation and its rarity [14, 15]. Furthermore, the majority of the published radiological literature consist of case reports. Most of the previous studies were on the diagnostic performance of computed tomography (CT) in the diagnosis of pancreatic schwannoma [3, 9, 16–20]. Although CT is often the primary imaging modality to use for the detection of pancreatic lesions, there is a growing trend in the total number of literature reporting the use of MR in the differential diagnosis of pancreatic neoplasms [13, 21–23]. However, there are limited prior studies to evaluate the role of diffusion-weighted imaging (DWI) and dynamic contrast-enhanced MR in the characterization of pancreatic schwannoma. In this study, we sought to discover the imaging features of pancreatic schwannoma on MRI from our case series and those from previous literature. To our knowledge, we report these largest multi-institutional case series regarding the MR-imaging features of pancreatic schwannoma that was previously recorded in the English and Chinese literature.

## Methods

The present study was performed in two parts.

The first part reported six patients from the histopathological and radiological database of First Affiliated Hospital of Fujian medical University (*n* = 1), Fujian medical university union hospital (*n* = 2), Fujian provincial hospital (*n* = 1), and Dongfang Hospital (*n* = 2) retrospectively, and the second part involved a literature review.

### Subjects

Research ethics board and local institutional approval from the four hospitals in the city were obtained for this retrospective study, and patient consent was waived. Multi-institutional pathology databases were searched over a 19-year period (January 1, 2000 to August 31, 2019), for histologically proven cases of pancreatic schwannoma. The corresponding preoperative MR images of these patients were likewise reviewed. All 6 lesions were surgically removed without any postoperative complications. The patients’ demographics, past medical history, clinical and all laboratory data, and operative documents were acquired from medical records. The follow-up information was also obtained from the patient or the treating physician.

The final our study group of six patients included five females and one male ranging in age from 25 to 57 years (mean, 46 years). Four (67%) patients presented with vague abdominal pain. Two (33%) patients were incidentally diagnosed on routine medical evaluation. Five patients (83%) had no smoking history. All six patients had no history of von Recklinghausen disease, pancreatitis, other pancreatic tumors, or pancreatic surgery. Four patients were evaluated by using dynamic enhanced CT as the first imaging modality. The remaining two patients were initially evaluated via both ultrasonography (US) and enhanced CT. However, both CT and US were difficult to provide a definitive diagnosis of pancreatic lesions with the nonspecific imaging features. All patients subsequently underwent further MRI examination. Interestingly, all of the present cases were initially misdiagnosed despite using US, CT, or MRI, including nonfunctional neuroendocrine in four cases, duodenal gastrointestinal stromal tumor in one case, and solid pseudopapillary neoplasm of the pancreas in one case. The final histopathological diagnosis of primary pancreatic schwannoma was established after pancreatic surgery. Patients’ characteristics were shown in Table 1.

**Table 1:** General demographics of study population

Present cases	Year	Age	Gender	Presenting Symptoms	Smoking Status	Surgical Procedure
Case 1	2019	25	F	Upper abdominal pain	Never	Pancreaticoduodenectomy
Case 2	2005	57	F	Periumbilical abdominal pain	Never	Pancreaticoduodenectomy
Case 3	2011	40	F	Abdominal pain	Never	Pancreaticoduodenectomy
Case 4	2016	55	F	Upper abdominal pain	Never	Pancreaticoduodenectomy
Case 5	2018	50	M	Asymptomatic	Former	Distal pancreatectomy
Case 6	2014	49	F	Asymptomatic	Never	Pancreaticoduodenectomy

### MRI examination

MR-imaging was performed with a 1.5 T scanner (two cases; Signa Excite; GE Medical Systems, Milwaukee, WI, USA) with a 8-channel phased-array coil, a 1.5 T scanner (one case; Magnetom Avanto; Siemens Healthcare, Germany), a 3.0-T imager (two cases; Magnetom Verio; Siemens Healthcare, Germany) with a 32-channel phased-array coil, and a 3.0-T imager with a 40-channel phased-array coil (one case; Siemens Skyra, Siemens Healthcare, Germany). The plain pancreatic MRI protocol on 1.5-T or 3-T MRI system included a free-breathing half-Fourier acquisition single-shot turbo spin-echo (HASTE) sequence, a T1 weighted dual fast gradient recalled echo sequence (in-phase and opposed-phase sequences), a breath-hold turbo spin-echo T2 weighted sequence, and free-breathing DWI with b values of (0,50) and (800,1000) s/mm^2^.

Dynamic contrast-enhanced MRI was performed with a fat-suppressed 3D gradient echo T1-weighted sequence (TR/TE = 4.0–5.4/1.3–4.0 msec, flip angle 9–20°, field of view 33-42 cm, matrix 128–192 × 256–512, slice thickness 3.0–5.0 mm). The arterial phase (25–35 s), portal venous phase (60–70s) and the delayed phase (200–240 s) images were obtained after intravenous injection of 0.1 mmol/kg gadolinium chelates (Gadobenate Dimeglumine Injection, Multihance (*n* = 2) or Magnevist (*n* = 4)) at a rate of about 2.5 ml/s.

### Systematic review

A literature search in PubMed, the China National Knowledge Infrastructure and Wanfang Database was conducted by two of the authors (Zhenshan shi and Zhongmin Li) on July 15, 2019, using medical subject headings term ‘pancreatic schwannoma’. We also searched the literature from the references of relevant documents to find other eligible articles. For the papers that were excluded because MR images were lacking, we attempted to communicate with the corresponding author to provide additional information. In total, the search yielded 196 potential articles; 34 articles written in language other than English or Chinese were excluded. Besides, 138 articles were excluded for further analysis because of lack of MRI examination or irrelevance. After excluding 172 references, 23 articles which included the MR images of pancreatic schwannoma were available for systematic review. Among them, 3 articles were written in Chinese. The period of publication ranged from March 1961 to July 2019.

#### Image interpretation

Two of the authors (X.M.L. and Z.M.L., abdominal radiologists with 15 and 12 years of experience in pancreatic MRI interpretation, respectively) independently determined the location, shape, size, margin, signal intensity (categorized as hypo-, iso-, or hyper-intense relative to normal pancreas), and enhancement pattern (subjectively defined as less than, equal to, or greater than normal pancreas, respectively) of the neoplasms on a picture archiving and communication system (PACS, Shida Co, Ltd., China). The MRI data were accessible through PACS, either performed in our institution or provided from referring institutions via compact discs. Disagreement between two observers was resolved by consensus. Both observers were blinded to the histopathologic and MR diagnosis of each case. ADC maps were generated automatically on the scanners. Regions of interest (ROIs) were drawn by consensus. Mean ROI size was 35.7 ± 15.4 mm^2^ (range, 15–63.8 mm^2^) and three repeated measurements of ADC values of the lesions were calculated. On the unenhanced T1-weighted MR images, the mean signal intensity (SI) value of three repeated measurements was recorded from the lesion and the nontumorous pancreas. SI ratios of lesion-pancreas were recorded by using the following formula: lesion-pancreas SI ratio = (SI lesion/SI pancreas).

### Pathology

All the hematoxylin and eosin (H&E)-stained tissue sections were reviewed retrospectively by an attending pathologist with expertise in pancreatic tumors to confirm the diagnosis of pancreatic schwannoma. All specimens have been fixed in 10% phosphate buffered formalin and paraffin-embedded. Immunohistochemical stains for pancreatic schwannoma including S-100, SOX10, Ki-67, DOG-1, C-kit protein (CD34, CD117), Desmin, PDGFR-a, SDHB, GFAP, P53, STAT6, EMA, and muscle-specific actin.

### Statistical analysis

Data analysis was conducted with SPSS statistical software, version 19.0 (IBM Corp., Armonk, NY, USA). The mean ± standard deviation (SD) was used to express continuous variables, while categorical variables were expressed as percentages and frequencies.

## Results

### MRI findings

**Fig. 1:**
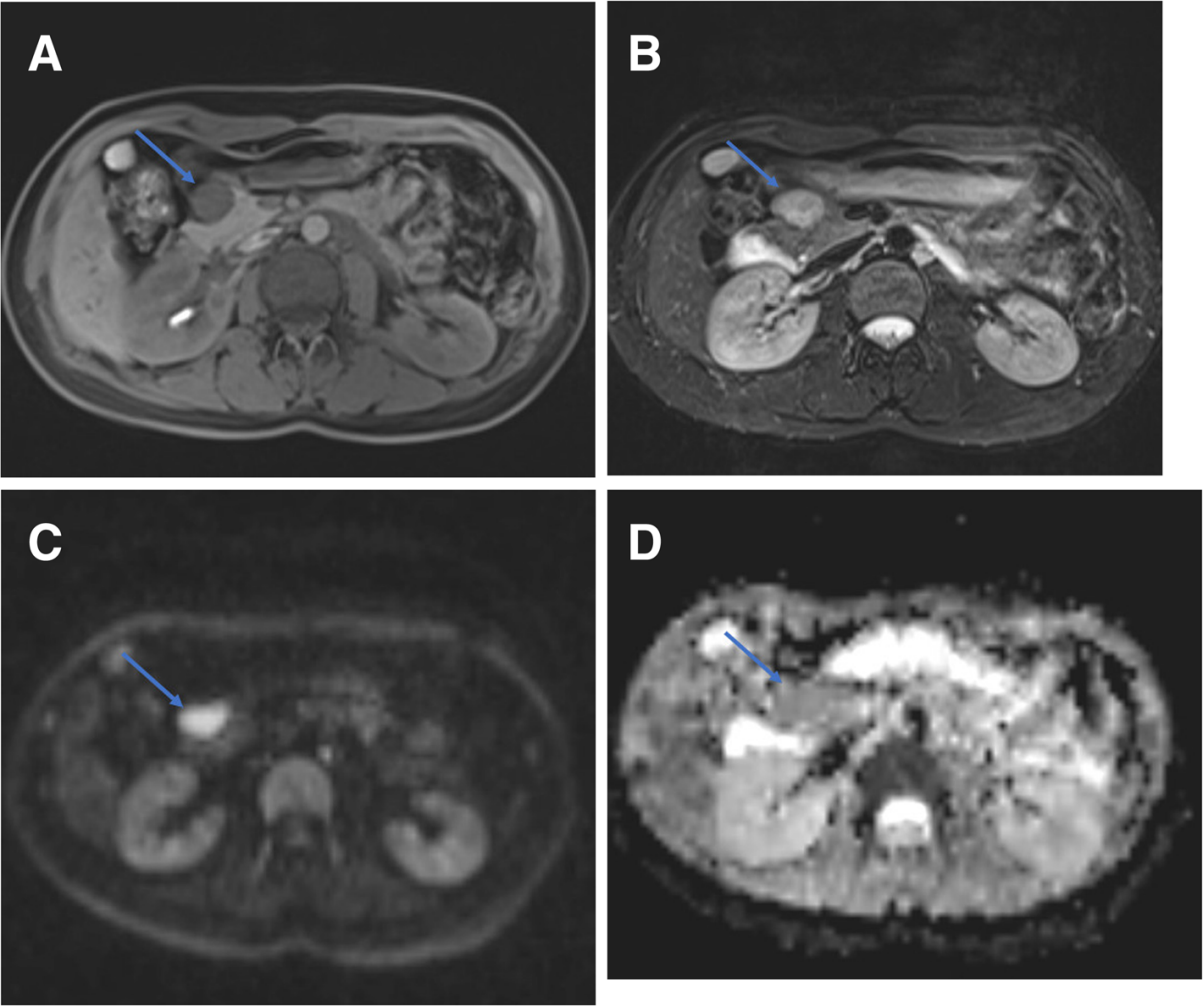
A well-circumscribed mass is seen in the head of the pancreas. The lesion is uniform hypo-intense on T1WI (**a**), homogeneous hyper-intense on T2WI (**b**), marked hyper-intense on DWI (b = 800 mm 2 /s) (**c**), and hypo-intense on ADC maps relative to the surrounding pancreas (**d**)

The MRI findings of pancreatic schwannoma are summarized in the Table 2. These lesions were solitary focus and appeared as a well-defined, oval or round mass with a median maximum size of 37 mm (range, 20-64 mm). Five tumors (83%) were located in the head-neck of the pancreas while one tumor was in the pancreatic tail. All pancreatic tumors showed hypointense on T1-weighted images and heterogeneously hyperintense on T2-weighted images compared with normal pancreas (Fig. 1a, b). No case had the imaging features of infiltration of adjacent structures, and calcified, hemorrhagic or prominent necrotic changes within the tumor on MR images. There was no succession between the pancreatic duct and the mass and no dilatation of the main or accessory pancreatic duct. No liver metastatic tumor or peripancreatic lymph node enlargement was detected.

**Table 2:** The summary of MRI findings

Pancreatic Schwannoma	Location			Boundary		Enhancement degree									SIR at unenhanced T1WI	ADC (×10^−3^ mm^2^/s)
	Head-neck	body	Tail	Well-defined	Ill-defined	Arterial phases			Portal phases			Delayed phases				
						Hyper	Iso	Hypo	Hyper	Iso	Hypo	Hyper	Iso	Hypo		
Number of cases	5	0	1	6	0	0	1	5	1	1	4	4	1	1	0.53 ± 0.07	1.11 ± 0.29

*SIR* Signal Intensity Ratio, *ADC* Apparent Diffusion Coefficient

**Fig. 2:**
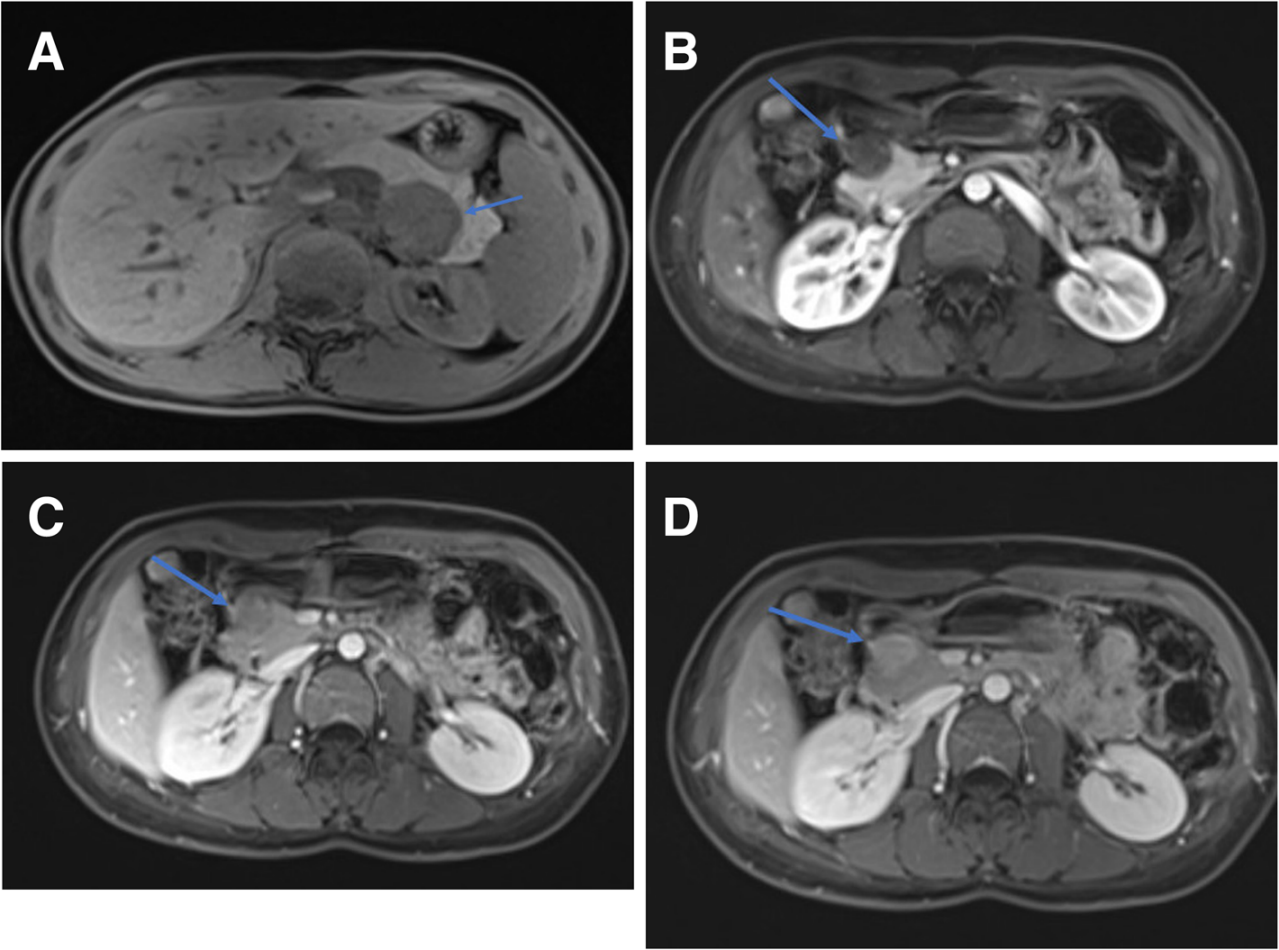
The pancreatic mass is illustrated on unenhanced and multiphase contrast enhanced images. The lesion shows uniform hypo-intense on unenhanced T1WI (**a**). The lesion exhibits gradual, homogeneous enhancement on dynamic contrast-enhanced MR images acquired at the arterial (**b**), portal (**c**), and delayed phase (**d**)

All tumors showed hyperintense on high b-value DW images and hypointense on the ADC maps relative to the surrounding parenchyma with whole-lesion ADC values ranging from 0.68–1.41 × 10^− 3^ mm^2^/s (Fig. 1c, d). The mean ADC value of pancreatic schwannoma was significantly lower than the surrounding pancreas. There was no area of signal loss on the in-phase or opposed-phase sequence and lesions remained hypointense (Fig. 2a). The signal intensity ratio of lesion-to-parenchyma on the unenhanced T1WI was 0.53 ± 0.07.

**Fig. 3:**
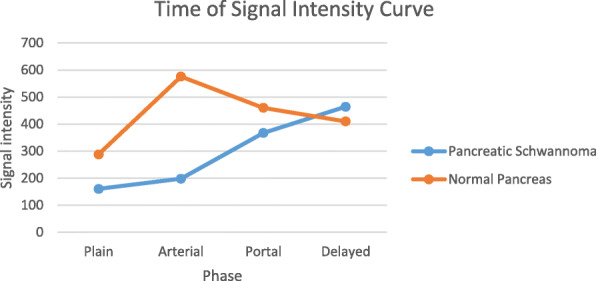
Time of signal intensity curve. The graph shows the time of signal intensity curve from dynamic contrast-enhanced magnetic resonance imaging of both the lesion and the normal pancreas

On dynamic contrast-enhanced images, five lesions (83%) demonstrated hypointense while one lesion (17%) showed isointense at the arterial phase. At the portal venous phase, four lesions (67%) showed hypointense while two lesions showed hyper- or isointense. At the delayed phase, four lesions (67%) showed hyperintense while two lesions showed hypo- or isointense. All six lesions showed early slight enhancement and persisted into the portal and delayed phases (Fig. 2b, c, d). The time of the intensity curve is shown in Fig. 3.

### Treatment

All cases were treated by surgical resection. Five patients (83%) underwent pancreaticoduodenectomy while one patient (17%) underwent a distal pancreatectomy. The median hospitalization time period was 13 days, and no patient developed severe postoperative complications.

### Pathologic findings

**Fig. 4:**
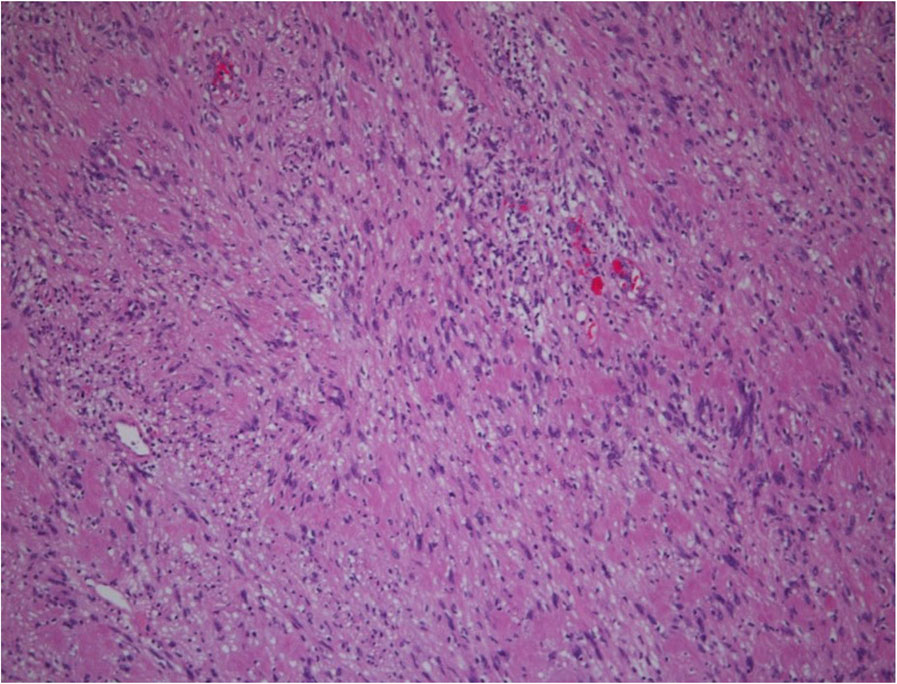
Microscopic examination. The pancreatic tumor shows spindle-shaped cells with a palisading arrangement of nuclei and no atypia. Hematoxylin and eosin stain; original magnification, 200×

Pathologic reports showed zones of closely packed spindle cells (Antoni A) and loose hypocellular region (Antoni B) with palisading Verocay bodies (Fig. 4). Immunohistochemistry showed that the pancreatic tumor cells were strongly positive for S100 (*n* = 6) and SOX10 (*n* = 2).

The ki-67 index was less than or equal to 5% and no mitosis was noted in all samples. Specimens also stained positively for CD34 (*n* = 3), PDGFR-a (*n* = 2), SDHB (*n* = 2), SMA (*n* = 1), GFAP (*n* = 1), P53 (*n* = 1,20%). Tumor cells showed negative staining for the following markers: CD117 (*n* = 4), CD34 (*n* = 3), DOG-1 (*n* = 4), STAT6 (*n* = 2), EMA (*n* = 2), Desmin (*n* = 3), and SMA (*n* = 3).

Follow up: The median follow-up period for all patients was 52 months (range: 6 to 124 months). Follow-up CT scan was available in six patients. Five patients are in regular checkups till now and have no clinical symptoms and no radiological signs of recurrence. One patient became lost to follow-up at 21 months so possible recurrent data are not available.

### Literature review

Multiple databases (PubMed, CNKI and Wanfang) search of the clinical literature with specific reference to the MR imaging features of pancreatic schwannoma indicated 23 reports including 25 patients in the English and Chinese literature. All of 25 previously reported cases, including patient age, patient gender, tumor location and size, and MRI features, are retrospectively reviewed.

There was a female predilection with a female to male ratio of 2.57:1 in the previous patients and the median age was 51.1 ± 13.2 years. The mass was most commonly situated in the pancreatic head (*N* = 17, 68%), while six tumors (24%) were located in the body of the pancreas and two tumors (8%) were located in the pancreatic tail. The median size of the tumor (reported on 23 patients) was 4.3 cm ± 2.2 (range 1.4-10 cm). On microscopic examination, most of lesions (*N* = 14) consisted of spindle cells with mixed Antoni A and Antoni B types, while only two lesions were composed of a predominantly Antoni A type and one lesion demonstrated Antoni B type. In accordance with published literatures and similar to our case series, a typical benign pancreatic schwannoma presented most commonly as a well-defined, oval or round mass with hypointense on T1WI, inhomogeneously hyperintense on T2WI, and a gradual enhancement pattern after gadolinium administration [3, 9, 13, 22]. No previous article had reported the use of DW-imaging and the ADC value in the diagnosis of pancreatic schwannoma because of its rarity, although the addition of DW-imaging can improve pancreatic lesion characterization, and generally results in a concomitant significant increase in the positive predictive value, sensitivity, and specificity of pancreatic MRI studies.

## Discussion

Pancreatic schwannomas, which were initially reported by Verocay [24] in 1910, are exceedingly rare and may be likely to associate with von Recklinghausen disease [2, 25–27]. Those patients with von Recklinghausen disease have an increased risk of malignant transformation and have a worse prognosis [2].

Pancreatic schwannomas are well-demarcated, encapsulated, homogeneous, tan-yellow, round solid masses on macroscopical examination [1, 3, 28, 29]. The classic features of pancreatic schwannomas often contain both hypercellular and hypocellular zones on microscopical examination [3, 4, 30]. Strongly positive immunohistochemical staining for SOX10 (*n* = 2) and S-100 (*n* = 6) were observed in our cases. The morphologic appearances of pancreatic schwannomas are virtually identical to those seen at other anatomic sites [31–35]. The uncommon anatomic site, rather than the distinct morphologic findings of the tumor, is the main source of difficulty in the preoperative diagnosis.

To date, only 25 cases have been reported in English and Chinese literature with particular reference to MR imaging findings. Together with our present case series, which is the largest single-institutional series to date, the total number of 31 patients with MR images have been identified.

Similar to the previous studies, the mean age of patients in our study was 46 years (range, 25-57 years) and most of the tumors were located in the pancreatic head. An interesting result observed in this study is a female predominance with a female: male ratio of 2.4:1, which is different from the earlier studies that revealed an almost equal sex distribution. The reason, which needs further investigation, may perhaps be a high female morbidity rate of pancreatic schwannoma in the Chinese population.

On MR imaging, pancreatic schwannomas appear as hypointense relative to the surrounding pancreas on T1-weighted images, and hyperintense on T2-weighted images. MRI appearances of the lesions in our case series are similar to the results of the previous literature. Additionally, hyperintense on DW imaging and hypointense on ADC maps were also seen in our series. However, there are limited data available to determine ADC threshold value in the diagnosis of pancreatic schwannoma.

Characteristic enhancement patterns of pancreatic schwannomas are mild heterogeneous/homogeneous enhancement at arterial phase and thereafter progressive enhancement at the portal and delayed phases, which are likely as a result of variable degree of pancreatic tumor cellularity or degenerative changes like cystic degeneration, calcification, hemorrhage, and necrosis [10, 36–38]. Although MRI findings of pancreatic schwannoma are usually similar to those of other abdominal sites (i.e. retroperitoneal, pelvic, hepatic, gastrointestinal tract) [6, 21], MRI may be useful in the preoperative differential diagnosis of pancreatic solid non-functional tumors.

In this context, the precise preoperative diagnosis of pancreatic schwannoma is generally difficult, and they are frequently confused with other pancreatic neoplasms, such as non-functioning endocrine neoplasms, solid pseudopapillary neoplasms, pancreatic ductal adenocarcinoma, and mucinous cystic neoplasms. It seems that the addition of MR imaging modality could not provide better diagnostic performance of preoperative diagnosis of pancreatic schwannoma in prior studies.

Based on our institutional experience and the pre-existing data, the presence of progressive hyperenhancement at the portal venous or delayed phase images and the higher enhancement degree than that of the surrounding pancreatic parenchyma at the delayed phase images seen in cases of pancreatic schwannoma would argue against pancreatic ductal adenocarcinoma, mucinous cystic neoplasms and solid pseudopapillary neoplasms. Furthermore, an ill-defined tumor boundary, metastatic lymph nodes, and the presence of upstream pancreatic ductal dilatation and parenchymal atrophy strongly suggests the diagnosis of adenocarcinoma. Imaging features of pancreatic schwannoma may overlap with non-functioning endocrine neoplasms; however, the lower mean lesion-to-parenchyma signal intensity ratio of 0.53 ± 0.07 on the unenhanced T1WI in our study may help in differentiating from the benign non-functioning endocrine neoplasms with the ratio of 0.64 ± 0.12 [39]. Nevertheless, it is not known whether the difference of the lesion-to-parenchyma signal intensity ratio between these two entities has the clinical and statistical significance, which will need further investigation.

Malignant transformation of schwannoma is rare, making up less than 1% of tumors and patients with von Recklinghausen’s disease are at increased risk of malignant schwannoma [25]. To some extent, malignant schwannoma shares basic imaging features with their benign counterpart. These imaging findings include a spindle-like shape and a predominantly longitudinal orientation in the nerve direction. However, some distinctions are quite noteworthy. Invasion of peripancreatic fat planes, large tumors (> 5 cm), unclear margins, heterogeneity, and perilesional edema are highly suggestive of malignancy. Malignant schwannoma is also more likely to recur and metastasize than benign or borderline tumor types [40–43].

In both our study and prior literature, all cases of resected pancreatic schwannoma achieved excellent long-term outcomes; none of the patients developed the recurrence during the last follow-up period. There was seemingly no difference in the choice of subsequent surgery type between those patients who underwent further MR imaging and those who did not in previous reports. As such, there remains a need to improve diagnostic imaging for precise diagnosis of pancreatic schwannoma.

This study has several limitations. The first limitation was the small number of patients due to the rarity of the tumor. Another limitation was the retrospective design, as with all retrospective studies, including the potential for incorrect data reporting and missed relevant studies for inclusion.

## Conclusions

In conclusion, a well-circumscribed mass displaying hypointense on T1WI, inhomogeneous hyperintense on T2WI, hyperintense on DWI, and gradual enhancement, suggests the diagnosis of pancreatic schwannoma. We consider that the MRI appearance plays a critical role in the accurate diagnosis of this disease. Despite its rarity, schwannoma must be discussed as one possibility in the list of differential diagnoses of pancreatic neoplasms.

## Data Availability

The datasets used and/or analyzed during the current study are available from the corresponding author on reasonable request.

## Abbreviations

T1WI: T1 weighted imaging
T2WI: T2 weighted imaging
DWI: Diffusion weighted imaging
ADC: Apparent Diffusion Coefficient
MRI: Magnetic Resonance Imaging
SIR: Signal intensity ratio
CT: Computed Tomography
HASTE: Half-flourier acquisition single-shot turbo spin-echo
VIBE: Volumetric interpolated breath-hold examination
ROIs: Regions of interest
SI: Signal intensity
H&E: Hematoxylin and eosin
MRCP: Magnetic resonance cholangiopancreatography
SD: Standard Deviation
US: Ultrasonography
EUS-FNA: Ultrasound-guided Fine-needle Aspiration
PET: Positron Emission Computed Tomography
ERCP: Endoscopic Retrograde Cholangiopancreatography
ETL: Echo train length
